# Optimising Golgi–Cox staining for use with perfusion-fixed brain tissue validated in the zQ175 mouse model of Huntington's disease

**DOI:** 10.1016/j.jneumeth.2015.09.033

**Published:** 2016-05-30

**Authors:** Zubeyde Bayram-Weston, Elliott Olsen, David J. Harrison, Stephen B. Dunnett, Simon P. Brooks

**Affiliations:** School of Bioscience, Cardiff University, Museum Avenue, Cardiff CF10 3AX, Wales, UK

**Keywords:** Golgi–Cox method, Paraformaldehyde, Section thickness, Huntington's disease, zQ175 mice

## Abstract

**Background:**

The Golgi–Cox stain is an established method for characterising neuron cell morphology. The method highlights neurite processes of stained cells allowing the complexity of dendritic branching to be measured.

**New methods:**

Conventional rapid Golgi and Golgi–Cox methods all require fresh impregnation in unfixed brain blocks. Here, we describe a modified method that gives high quality staining on brain tissue blocks perfusion-fixed with 4% paraformaldehyde (PFA) and post-fixed by immersion for 24 h.

**Results:**

Tissue perfused with 4% PFA and post fixed for 24 h remained viable for the modified Golgi–Cox silver impregnation staining of mouse striatum from perfused wild type and zQ175. It was not found necessary to impregnate tissue blocks with Golgi solutions prior to sectioning, as post-sectioned tissues yielded equally good impregnation. Impregnation for 14 days resulted in optimal visualisation of striatal neuron and dendritic morphology. Although no modifications applied to the rapid Golgi method were reliable, the modified Golgi–Cox method yielded consistently reliable high-quality staining.

**Comparison with existing methods:**

The current method used fixed tissues to reduce damage and preserve cell morphology. The revised method was found to be fast, reliable and cost effective without the need for expensive staining kits and could be performed in any neuroscience lab with limited specialist equipment.

**Conclusions:**

The present study introduces a robust reproducible and inexpensive staining method for identifying neuronal morphological changes in the post fixed mouse brain, and is suitable for assessing changes in cell morphology in models of neurodegeneration and in response to experimental treatment.

## Highlights

• A modified reliable Golgi–Cox method is described.
• It is inexpensive and can be performed in any neuroscience lab.
• Perfusion–fixation with 4% paraformaldehyde yields good results.
• Pre-sectioning of perfusion-fixed brain tissue is neither required nor preferred for modified Golgi–Cox staining.
• We demonstrate reliable changes in striatal dendritic morphology in the Q175 mouse model of Huntington's disease.

## 1 Introduction

Camillo Golgi discovered a basic method for visualising neurons in the nervous system in 1873, which was originally named the black reaction (*la reazione nera*). The Golgi technique stains only 3–5% of cells in the brain, at random and by unidentified mechanism (Spacek, 1989). Golgi revealed that his procedure of nervous tissue hardening took place after incubation in potassium dichromate, followed by impregnation with silver nitrate (Golgi, 1873). Cox (1891) modified the original technique by replacing the silver nitrate, responsible for the impregnation of neurons, with mercuric chloride and termed Golgi–Cox (Cox, 1891, Stean, 1974).

Thus depending on the type of salt used, Golgi staining is subcategorised into two major types: those producing a deposit of silver are referred to as “rapid Golgi”, those causing a deposit of mercury as “Golgi–Cox”. Golgi's staining technique is selective in allowing visualisation of neurons in their entirety including cell soma, axons, dendrites, and spines. This allows researchers to examine, characterise and quantify axonal and dendritic morphology and spines throughout the nervous system (Risher et al., 2014). 140 years after its introduction, Golgi staining remains one of the most powerful methods for use in quantitative structural neuroanatomy, and allows for evaluation of the relationships between brain morphology and behaviour (Gibb and Kolb, 1998, Ivy et al., 2010, Williams et al., 1980). The staining methods have been adapted and improved within the field over the time for newer approaches, such as introduction into electron microscopy and combination with immunohistochemistry and pathway tracing (Buller and Rossi, 1993, Orlowski and Bjarkam, 2012, Ranjan and Mallick, 2010, Spiga et al., 2011, Zhang et al., 2011). However, in human post-mortem tissue, the technique has some drawbacks, in particular: (i) inconsistency of staining (Buell, 1982); (ii) lack of uniformity (Pasternak and Woolsey, 1975); (iii) the requirement for long immersion times of tissue blocks for effective impregnation (Glaser and Van der Loos, 1981); and (iv) the need for a vibrotome for sectioning unfixed tissues (Narayanan et al., 2014).

To address the potential effectiveness of this approach we sought to develop a stable, reproducible and uniform modified Golgi method that permits rapid Golgi or Golgi–Cox impregnation of brain tissues fixed with 4% paraformaldehyde and microtome sectioning, in contrast to the use of unfixed tissues and consequent vibratome sectioning of existing methods. To address the reproducibility problem we evaluated different impregnation protocols using silver and mercury salts applied either to fixed tissue blocks prior to sectioning and to free-floating sections. To determine the optimum tissue thickness, brains were cut at 80 μm and 120 μm and analysed using light microscopy. All procedures were carried out using half brains allowing the remaining tissue to be used for other experiments, and thereby reduce the number of animals used.

To validate the utility of the method we evaluated morphological changes in the striatal neurons of zQ175 HD mouse against wild type litter mate controls in this knock-in genetic model of Huntington's disease (HD). HD is a neurodegenerative disease characterised by neuronal cell loss primary within the caudate nucleus (Vonsattel et al., 1985). The study of Golgi stained human autopsy material has provided evidence for proliferative and degenerative changes in dendritic spines within post-mortem HD striatum (Ferrante et al., 1991, Graveland et al., 1985a, Graveland et al., 1985b, Sotrel et al., 1993), and in mouse models (Guidetti et al., 2001, Heck et al., 2012, Klapstein et al., 2001, Nithianantharajah et al., 2009). It is plausible that early stage subtle cognitive changes are caused by disruption of corticostriatal synaptic communication reflected in changes in striatal dendritic morphology. Consequently, the zQ175 mouse model, we assessed at an age when cognitive changes are first becoming apparent (Harrison DJ and Brooks SB unpublished data).

## 2 Materials and methods

### 2.1 Animals

A total of 32 mice were used for the study. Animals were kept on a 12 h/12 h light–dark cycle (lights on 06:00 hour) with ad libitum access to food and water and an ambient room temperature of 21 ± 1 °C. All experiments were conducted in accordance with the UK Animals (Scientific Procedures) Act 1986, and local ethical review.

Sixteen (*n* = 7 male, *n* = 9 female) C57BL/6J mice (Harlan, UK) were used to optimise the working protocol at 4 months of age. The final working procedure was applied to on additional 16 mice (8 heterozygous and 8 littermate wild-types; 8 male/8 female) of the zQ175 strain, assessed at 12 months of age. The Q175 strain was originally purchased from Jackson Laboratory (Bar Harbour, USA) and the colony was maintained in house by back crossing onto a C57BL/6J background (Harlan, UK) over six generations. The mice were genotyped commercially by tail tipping and DNA extraction (Laragen Inc, Los Angeles, USA) and the 8 experimental mice exhibited a CAG repeat length 194 ± 14. All mice were housed in mixed genotype, single-sex cages under standard animal laboratory conditions.

### 2.2 Histological methods

#### 2.2.1 Golgi stain optimisation

We used a modified Golgi staining method adapted from the protocols used by Wright et al. (2011) and sought to optimise a number of the methodological steps.

##### 2.2.1.1 Preparation of Golgi Stock solutions: Rapid Golgi–Cox vs. Golgi–Cox

Two different base solutions were prepared and applied for Golgi staining:

###### 2.2.1.1.1 Golgi solution with silver nitrate (rapid Golgi)

The rapid Golgi impregnation used two solutions as follows:

*Solution A.* 3% Potassium dichromate solution (Sigma-Aldrich, no. P5271, USA) stirred into warm deionised water.

*Solution B.* 2% Silver nitrate solution (Sigma-Aldrich, no. 20,913-9, Germany) stirred into deionised water.

These solutions are never mixed with each another.

The tissues were placed 3% potassium dichromate Solution for 7 days in the dark, changing solutions daily using glass pipettes, and then transferred into 2% Silver Nitrate Solution for 3 days in the dark at room temperature.

###### 2.2.1.1.2 Golgi solution with mercuric chloride (Golgi–Cox)

The Golgi–Cox Solution consists of two solutions as follows:

*Solution A.* 100 ml of 5% potassium dichromate solution (Sigma-Aldrich, no. P5271, USA) stirred into warm deionised water, with 100 ml 5% mercuric chloride (Sigma-Aldrich, no. M136, India) stirred into hot deionised water.

*Solution B.* 200 ml of dH_2_O and 80 ml of 5% potassium chromate (Sigma-Aldrich, no. 216615, USA) stirred into cold deionised water.

Solution A was then slowly poured into solution B with constantly stirring. When mixed correctly, a red yellow precipitate is formed.

The Golgi–Cox solution was stored in the dark for 72 h, and then filtered before use.

##### 2.2.1.2 Tissue fixation

Most studies have applied Golgi impregnation on fresh tissues rather than the fixed tissues. Here, we have applied the impregnation process on both unfixed and fixed tissues with 0.9% saline, (no fixation), 1.5% paraformaldehyde in 0.1 M PBS (1.5% PFA) and 4% paraformaldehyde in 0.1 M PBS (4% PFA) (Fischer Scientific, Loughborough, UK).

Briefly, the Blk/6 mice were killed by intraperitoneal injection of 0.2 ml Euthetal (Merial, Essex, UK) and then perfused transcardially with 0.9% of saline for 3 min, followed by either 0.9% of saline, 1.5% PFA or 4% PFA in for a further 5 min. After perfusion, of the 16 brains used 2 remained in 0.9% saline, 2 in 1.5% PFA and 2 for 4% PFA for 1hr to attempt to replicate and optimise previous studies. As these short perfusion time failed to work, the remaining 10 brains were immersed in 1.5% PFA (*n* = 5) or 4% PFA (*n* = 5) for 24 h at room temperature, respectively as per our standard tissue fixation procedure.

##### 2.2.1.3 Free floating sections versus half brain processing

As a next step, we applied two paths to test the feasibility of using the pre-cut sections for Golgi staining. The first path was to cut tissue blocks into coronal sections and then impregnate alternating series in the rapid Golgi and Golgi–Cox solutions. Half of the hemispheres were placed into cryoprotectant 25% sucrose/PBS solution for 24 h and then both 80 μm and 120 μm sections were cut, (1:6 series), using a freezing-stage sledge microtome (Leitz Bright Series 8000, Germany). Sections were incubated in Silver (rapid Golgi) or mercury (Golgi–Cox) based solutions for 10 days or 14 days in the dark. The sections were then transferred into Petri dishes containing 0.1% Triton X-100 in 0.1 M Tris-buffered saline (TXTBS) solution for washing mounted on gelatine-covered slides and air-dried. The silver nitrate impregnated sections had an unexpected reaction with TXTBS solution; they proved extremely difficult to mount on gelatine-coated slides and the sections fragmented on drying. TBS and dH_2_O were tried as alternative washing solutions, but with the same adverse effects on tissue integrity.

As the above procedure failed, a second path was tested using different perfusions methods (1.5% PFA and 4% PFA) and was more successful. After each perfusion, brains were bisected along the midline, with half of the brain transferred into Silver (rapid Golgi) solution without sectioning for 10 days, and the other half into mercury (Golgi–Cox) based solution without sectioning for 7 days, 10 days or 14 days in the dark. After sufficient impregnation time, excess liquid was then removed using blotting paper, before placing the hemispheres into a solution of 25% sucrose in PBS where they stayed until they equilibrated and sank. Blocks were then cut coronal sections at 80 μm thickness, in a 1:6 series, using a Leica freezing-stage sledge microtome. Sections were collected in a TBS buffer solution at +4 °C, and mounted onto gelatine-coated glass slides.

The optimal protocol that we identified required 24 h post fixation of the brain hemispheres and use of the Golgi–Cox solution. Although all incubation time points resulted in some staining of the tissue, 14 days incubation yielded best staining.

### 2.3 Golgi–Cox staining of zQ175 mice

The modified protocol was applied for comparing the morphology of striatal dendritic morphology in zQ175 HD mice with that of their wild type littermates. Briefly, 12 month old zQ175 mice were killed by intraperitoneal injection of 0.2 ml of Euthetal and then perfused intracardially with 0.9% saline for 3 min, followed by 4% PFA in a 0.1 M PBS solution (pH 7.4) for a further 5 min. The brains were then immersed in 4% PFA overnight at room temperature and then cut into two hemispheres. The right hemispheres were transferred into 25% sucrose in PBS until they sank. The left hemispheres were transferred into Golgi–Cox solution for 14 days in the dark, with the solution changed daily. The excess liquid was then removed using blotting paper, before placing the brains into 25% sucrose in PBS and stored in the dark until they sank. The brains were then cut into 80 μm coronal sections using a freezing-stage sledge microtome. Cut sections were collected in a 1:6 series in a TBS solution at +4 °C. Sections were mounted onto gelatine-coated slides and left to air dry overnight at room temperature in the dark. The following day, sections were washed with dH_2_O for 5 min and transferred into 20% ammonium hydroxide with dH_2_O (Sigma-Aldrich, no. 320145, USA) for 10 min. The sections were washed in dH_2_O for a further 5 min, passed through ascending grades of alcohol (70%, 95% and 100%) and placed in xylene for 40 min, followed by cover slipping with DPX (Thermo Scientific, UK) medium.

### 2.4 Stereological analysis

Striatal cells quantification was determined by two-dimensional stereology using an Olympus BX50 microscope (Olympus Optical Co., Ltd., Tokyo, Japan), utilising PC based image analysis software (Olympus C.A.S.T. grid system v1.6). A meander sampling methodology was used to avoid bias in the selection of neurons throughout the striatum that showed complete Golgi impregnation. For each slide striatal sections were outlined under a 1.25× objective lens. Defined striatal sections were then sampled at random. The sampling step length was set to 500 μm, in order to allow a sample size large enough for the required number of neurons to be examined (approx. 10 neurons per section over 5 to 6 sections per animal). With the microscope set at a magnification of 400×, if the neuron cell body fell within the sampling frame, the neuron would be accepted and analysed. If it was outside the frame, the stage was moved to the next sample. The parameters being analysed were: cell diameter; dendritic arborisation (number of branches); dendrite length; number of spines on the dendrite; number of spines (per μm of dendrite); spine maturity as defined by spine shape (Irwin et al., 2002, Risher et al., 2014).

**Fig. 1:**
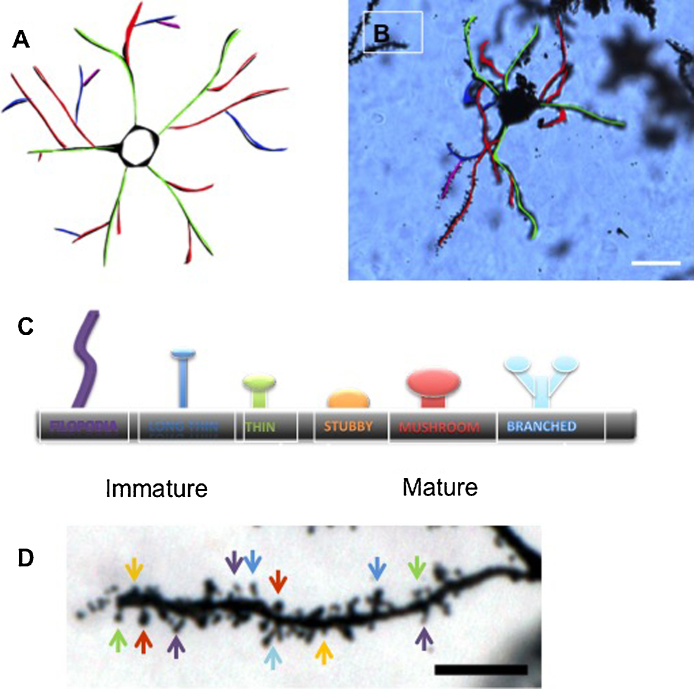
Dendritic branching of medium spiny neurons. (A) An illustration to show the levels of complexity of dendritic branching of medium spiny neurons found within the striatum. The branches correspond to the following colours; primary (green), secondary (red), tertiary (blue), quaternary (purple). (B) A medium spiny neuron in the striatum of a zQ175 mouse. Colour-coded to match (scale bar = 20 μm). (C) An illustration to show the range of dendritic spines that are common when examining the dendrites of medium spiny neurons within the striatum of zQ175 mice. The spine maturity level increases from left to right along the diagram, with filopodia (purple) being the least mature and branched (light blue) being the most mature. (D) Golgi–Cox stained dendritic branch of a medium spiny neuron of the zQ175 striatum. Different spine types are indicated by arrows, colour-coded to match (scale bar = 5 μm). (For interpretation of the references to colour in this figure legend, the reader is referred to the web version of this article.)

Cell diameter was measured using the digital straight ruler tool, with values taken for both the horizontal and vertical diameters. These numbers were then averaged to give an average cell diameter. The analysis of branches was broken down into primary, secondary, tertiary and quaternary branches as defined by the number of branches away from the cell body (Fig. 1A and B), with each level counted and recorded separately for each neuron. Dendrite length was measured using the digital curve length ruler tool in the software programme, with measurements taken from the base of the dendrite at the neuron body, to the tip of the dendrite.

The number of spines was then counted on the entire dendrite for which the value for dendritic length had been obtained, and the number of spines per μm of dendrite was determined. Spine maturity was classified in order to determine if maturity was affected when comparing the wildtype and heterozygote mice. The morphological classifications were based on Irwin et al. (2002), and allocated to 6 maturity levels of spine morphology using the shapes in Fig. 1C and D, (adapted from Risher et al., 2014). The Straight Ruler Tool was used to measure width or diameter of the spine to determine maturity, with of all the spines on the dendrite measured and recorded. The mean of all neurons in each individual animal was used for statistical comparison between wild type and knock in mice.

### 2.5 Statistical analysis

Parametric between-subjects or split-plot analysis of varience (ANOVA; Genstat for Windows v.16.2) and independent *t*-tests were used to determine genotype effects on the measures applied, as appropriate.

## 3 Results

### 3.1 Comparison of silver nitrate and mercuric chloride stains in pre-sectioned brain tissue

Silver nitrate sections placed into TXTBS and TBS caused widespread accumulation of metal staining that rendered the sections unusable. The sections were also brittle and unmountable. If the sections were taken directly from the silver nitrate solution without washing in TXTBS they could be mounted on gelatin slides but disintegrated whilst drying. When tested on un-sectioned intact brains no neurons were found to be stained.

By contrast, Golgi–Cox based impregnation using mercuric chloride solutions was more successful. In pre-sectioned brains no neuronal staining was found, and in brains perfused with 0.9% saline or 1.5% PFA and post fixed for 1 h, the tissue collapsed when cutting on the microtome. However, when fixed using PFA at 4% and a perfusion time of 24 h, the tissue was more durable and could be easily processed. Unlike silver impregnation, there were no issues with using TXTBS and TBS post-staining. This method showed good staining of neurons throughout the brain. We then sought to determine the optimal Golgi–Cox incubation period.

**Fig. 2:**
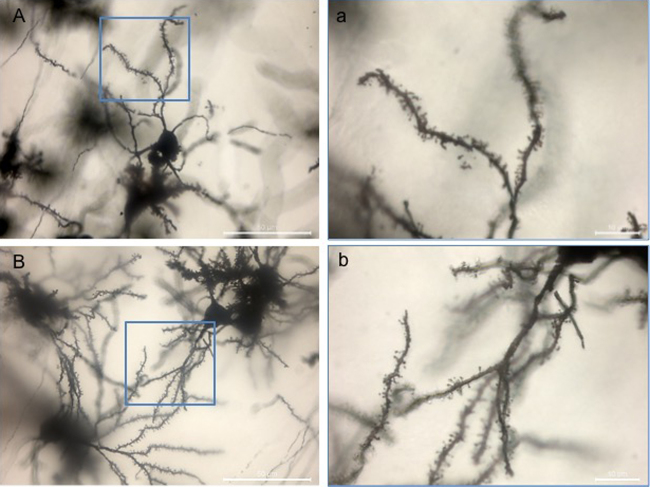
Golgi–Cox staining of medium spiny neurons in the striatum of the Q175 mouse line. (A) The dendritic spines and branching of a medium spiny neuron in the striatum are clearly visible in the wild type zQ175 mouse (scale bar = 50 μm). (a) With the higher magnification it is easy to focus on the different spines to determine maturity, as the stain is sharp and clear (scale bar = 10 μm). (B) Good staining again of a medium spiny neuron, with the complexity of dendritic branching easy to see within the striatum of the Het zQ175 mouse (scale bar = 50 μm). (b) The different levels of spine maturity were clearly visible in the Het mice (scale bar =10 μm).

**Fig. 3:**
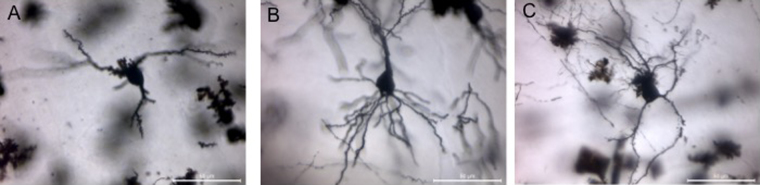
Golgi–Cox staining of different type neurons in the different regions of the Q175 mouse line. (A) Cortex, (B) hippocampus and (C) the purkinje cell of the cerebellum (scale bar = 50 μm).

The Golgi–Cox impregnated neurons with detailed dendritic trees were clearly visible at 12 months of age in the striatum of the zQ175 HD mice and their littermate (Fig. 2A and B). Those detailed dendritic trees were also visible in other regions of the brain including the cortex, hippocampus and cerebellum at this time point (Fig. 3).

### 3.2 Optimising the Golgi–Cox incubation period

The optimum incubation time in the Golgi–Cox stain was determined by testing 7, 10 and 14 day periods. At 7 days there were low levels of staining seen throughout the brain. Slight improvement was seen at 10 days with neuronal bodies, coupled with small lengths of dendrite being stained. By 14 days detailed dendritic trees could be viewed. Morphologically, there were no differences between the 80 and 120 μm sections; however, 120 μm displayed mild background staining, therefore we sectioned at 80 μm afterwards. After storage in TBS at +4 °C for 2 mo. after being cut, 1/12 series were stained and still displayed good neuronal morphology.

As a result the following parameters were accepted for our refined protocol; perfusion for 24 h in 4% PFA, half hemisphere brain blocks impregnated with mercuric chloride solution for 14 days, cut at 80 μm section thickness and stored, refrigerated in TBS.

### 3.3 Validation of the optimisation procedure in the zQ175 mouse line

**Fig. 4:**
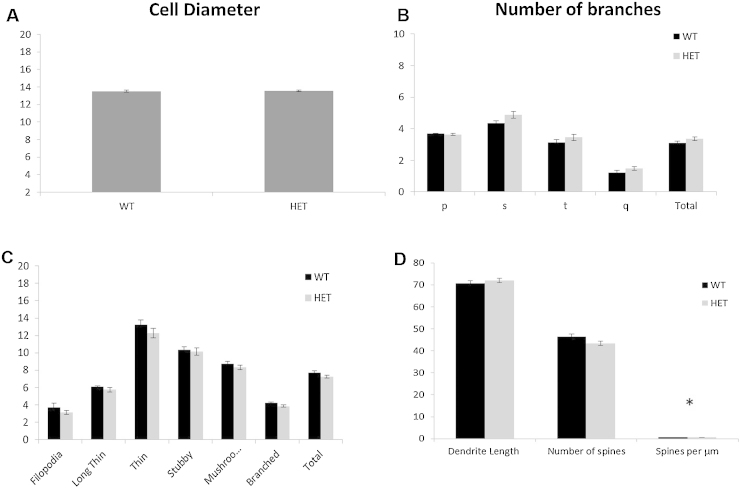
(A) A graph to show the comparison in cell diameter between the wild type and Het groups of the zQ175 mice. (B) A graph to show the difference in the number of primary, secondary, tertiary and quaternary branches between the wild type and Het groups of the zQ175 mouse line. The means of the number of overall branches are also compared. Spine morphology categories. (C) A graph to compare the number of spines at each maturity level and the number of spines overall in between the wild type and Het groups of the zQ175 mouse line. (D) A graph to compare the following parameters between the wild type and Het groups of the zQ175 mice; the complexity of dendritic branching, the number of spines per dendrite and the number of spines per μm. Error bars are S.E.M.s.

We then validated this approach using a stereological analysis of striatal-neurone morphology in the zQ175 HD mouse line. Cell bodies were readily identified and measurable (Fig. 1, Fig. 2) and there was no suggestion of any differences in cell diameter between the wild-type and heterozygote zQ175 mice (Fig. 4A: *F* × *t*_14_ = 0.074, n.s.). Similarly, dendrites and dendritic trees were visible and amenable to quantification. Whilst the dendritic trees were highly visible under light microscopy no differences were found in the lengths of dendritic branches between the genotypes (*t*_14_ = 0.30, n.s.), nor in the degree of arborisation and the number of primary, secondary, tertiary or quaternary branches (Fig. 4B: *F*_3, 42_ = 1.93, n.s.). The dendritic spines were clearly visible under light microscopy and could be classified by shape in to their maturation stages, but no differences between genotypes were found (Fig. 4C: *F*_5, 70_ = 0.47, n.s.). However, overall, the zQ175 mice did demonstrate a decrease from wild-type levels in the density and number of spines/μm (Fig. 4D: *t*_14_ = 2.41, *p* < 0.05).

### 3.4 Discussion

This study describes a simple Golgi staining protocol designed to be used with perfused and post-fixed brain tissue. The rationale behind the modified protocol was to reduce the animal numbers and increase the ability to obtain multiple measurements within the individual animal in a single experiment. We found the rapid Golgi derived silver impregnation method was less effective than anticipated but that a Golgi–Cox derived mercuric chloride procedure did yield robust results. This observation is compatible with previous studies that have also found the silver stains to be less predictable (Friedland et al., 2006, Gabbott and Somogyi, 1984, Pasternak and Woolsey, 1975). Here we have shown that our modified protocol produced clear images under light microscopy that could be used reliably and efficiently for measuring cellular morphology in detail in fixed brain tissues.

Staining artefacts are common with these procedures and are thought to arise through a non-selective reaction between potassium dichromate and silver nitrate, causing bulk crystals to form on the surface of the specimen (Pasternak and Woolsey, 1975). In the present study artefacts were also noted but using the coronal sections on the half-brain units still provided a high number of cells from which to take random samples with stereology. In addition, this method was inexpensive and easy to carry out.

Wright et al. (2011) demonstrated the use of no fixative when staining the brain, with other methods experimenting with osmium tetroxide, which was found to stabilise the cell membranes (de Castro et al., 2007). Others (Colonnier, 1964, Fox et al., 1951) have modified the Golgi method to allow staining of formalin-fixed brains with further modifications coupling formalin with dichromate salt and chloral hydrate and using glutaraldehyde. However in these studies, a vibratome was still used throughout to obtain good sections. We found the best fixative to perfuse the brain in situ was 4% PFA with a post-fixation for 24 h prior to submergence in the Golgi–Cox solution. This procedure allowed the brains to be easily sectioned with a sledge microtome. Even though half brains in coronal sections were used, the most effective incubation time was 14 days as found in the Wright et al. (2011) study that used whole brains.

Other studies have previously suggested that enhanced visualisation of dendritic spine morphology can be achieved through sectioning the brains pre-staining when using the Golgi–Cox method (Landas and Phillips, 1982, Levine et al., 2013). Golgi impregnation has been used successfully on pre-fixed (10% formalin) and sectioned (100 μm) tissue (Ferrante et al., 1991, Landas and Phillips, 1982). Similarly, it has been successfully shown in rat brains that 100 μm pre-sectioned tissue perfused with 0.9% saline also worked well (Levine et al., 2013). However our study is in agreement with others (Friedland et al., 2006), in finding that pre-sectioning of the tissue resulted in poor neuronal staining. This may be because by the neurons need be intact prior to staining in order to allow best penetration of the impregnation solution, but this is in effect an empirical decision in the light of the still unexplained nature of the Golgi reaction.

Section thickness used in previous Golgi studies typically varies between 40 and 300 μm, with some authors suggesting that thicker sections can be used if material clarity permits (Gabbott and Somogyi, 1984, Glaser and Van der Loos, 1981, Landas and Phillips, 1982). Good neuronal profiles have been shown in sections between 60 and 200 μm (Gabbott and Somogyi, 1984). One study has even suggested that sections of a thickness greater than 300 μm can be also used and stained (Glaser and Van der Loos, 1981). Our results on a relatively narrow range of sections found no obvious differences between viewing dendritic trees and dendritic spines in 80 μm and 120 μm sections. It was decided that 80 μm would be used in our protocol as more sections can be made available for further staining and analysis, the disadvantage is that whole intact dendritic trees may be more difficult to find in thin slices. However, in our 120 μm sections it was found that neurons and dendrites overlapped, making it more difficult to distinguish dendrite complexity and spines.

When the present modified method was applied to the zQ175 mouse lines, clear images of cells were produced that were amenable to quantification of a full range of morphological features. Dendritic arborisation and cell spines types were clearly visible and reliably quantifiable under light microscopy. In the HD mouse line used, only the density of spines/μm of denrite was found to be signficantly reduced in the heterozygotes which is in agreement with other HD mouse (Klapstein et al., 2001, Spires et al., 2004) and patient studies (Heck et al., 2012, Klapstein et al., 2001, Sotrel et al., 1993). Changes in dendritic branching have been reported previously in HD mouse lines (Ferrante et al., 1991, Madison et al., 2012), with other authors finding no differences between genotypes (Spires et al., 2004) as in the present study. As most studies in HD mouse lines focus on the striatum it may be that there is regional specificity to any effects that may yet be identified as found previously (Nithianantharajah et al., 2009). These differences may also reflect the different mouse models being used.

## 4 Conclusions

The modified staining protocol described in the present study has advantages over previous procedures: (i) it works on half-brains permitting additional analysis with the remaining hemisphere and reduction of animal numbers used; (ii) brains can be cut with a microtome eliminating the need for expensive equipment; (iii) the protocol is easy to follow allowing stains to be done in any lab; (iv) it has minimal artefacts compared to ‘rapid Golgi’ methods; (v) it is inexpensive.

## Figures and Tables

**Table:** 

TRIS base (Sigma-Aldrich, no. T6066, USA)	48 g
Sodium chloride (Sigma-Aldrich, no. 71376, USA)	36 g
Distilled water	1000 ml
Adjusted to pH7.3 with concentrated HCl and stored at +4 °C	

**Table:** 

Sucrose (Fischer Scientific, no. S/8600/60, UK)	25 g
TBS	Make up to 100 ml

## Appendix A

### A.1 Buffers and solutions

#### A.1.2 TRIS buffered saline II—0.1 M (Working solution)

250 ml of stock solution (above) +750 ml Distilled water Adjusted to pH 7.3 with concentrated HCl and stored at +4 °C.

#### A.1.3 TX-TBS

250 ml of working solution of TBS + 500 μl Triton X-100 (Sigma-Aldrich, no. X100, USA)

Once dissolved: pH to 7.4 with concentrated HCl and stored at +4 °C

#### A.1.4 4% Paraformaldehyde solution in 0.1 M Sorensen buffer

16gr PFA (Fischer Scientific, Loughborough, UK) in 150 dH_2_O with couple drops of NAOH put on the stirrer and heat to 60 °C approx. 30 min and add 50 ml dH_2_O = 200 ml of 8% PFA (filter the solution)

200 ml of 8% PFA (filtered) + 200 ml prewash buffer = 400 ml of 4% PFA

#### A.1.6 Golgi–Cox solution

*(SolA) 5% potassium dichromate solution.* 5 g Potassium Dichromate (Sigma-Aldrich, no. P5271, USA) stirred into 100 ml warm deionised water.

*(SolB) 5% mercuric chloride solution.* 5 g Mercuric Chloride (Sigma-Aldrich, no. M136, India) stirred into 100 ml hot deionised water.

*(SolC) 5% potassium chromate solution.* 5 g Potassium chromate (Sigma-Aldrich, no. 216615, USA) added to 100 ml cold deionised water. Continue to stir until dissolved.

100 ml of Sol A was then added to100 ml Sol B. Following this, 200 ml of dH_2_O and 80 ml of Sol C were added together. The Sol A–B mixture was then slowly poured into the diluted C solution while constantly being stirred until dissolved. When mixed correctly, a red yellow precipitate is formed. The Golgi–Cox solution was stored in the dark for 3 days, followed by filtering before use.

### A.2 Golgi–Cox protocol

(1) Animals were perfused with 0.9% Saline for 3 min and than with 4% PFA for 5 min.
(2) Remove the brains out and post fixed in 4% PFA for 24 h.
(3) Remove the brains from the 4% PFA solution and place it in Golgi–Cox solution for 14 days.
(4) Remove the brains from the Golgi–Cox solution. Using blotting paper, remove excess liquid before placing the brain into 25% sucrose.
(5) Wait for the brains to sink.
(6) Cut the brains into 80 μm sections and store in TBS.
(7) Mount the sections onto gelatin slides, using TBS and TXTBS and a flotation medium.
(8) Let them air dry for 24 h.
(9) Wash the sections with dH_2_O for 2 min.
(10) Incubate the sections in 20% ammonium for 10 min.
(11) Wash sections in dH_2_O for 2 min.
(12) Go through descending ethanol series 70%, 95%, 100% (5 min each) and 2× xylene (10 min each).
(13) Coverslip with DPX (Thermo Scientific, UK) medium.
